# ZASCA-sum: A dataset of the South Africa supreme courts of appeal judgments and media summaries for legal documents summarization research

**DOI:** 10.1016/j.dib.2025.111567

**Published:** 2025-04-19

**Authors:** Idris Abdulmumin, Vukosi Marivate

**Affiliations:** Data Science for Social Impact Research Group, Department of Computer Science, University of Pretoria, Lynnwood Road, Pretoria 0028, Gauteng, South Africa

**Keywords:** Natural language processing, Document summarization, Legal summarization, Summarization corpora, Supreme Court of Appeal of South Africa

## Abstract

This paper presents ZASCA-Sum, a novel dataset comprising judgments from the South Africa Supreme Court of Appeal and their manually curated media summaries. The dataset, collected from the court's official website, includes 4171 judgments, of which 2118 have summary pairs. The judgments and summaries have been extracted and prepared to support legal document summarization tasks across supervised, semi-supervised, and unsupervised settings. This paper provides a detailed description of the dataset, covering the data collection process, timeline, processing, and potential applications in the field. We provide the token-count distribution and analysis of the judgments and summaries that can be accommodated off-the-shelf by current summarization models with the largest input token size. The dataset, split into training, validation, and test sets, is made publicly available to encourage research in legal summarization. In addition to document summarization, researchers can use this data to localize English-centric models to support the South African dialect.

Specifications Table

**Table:** 

Subject	Computer Science
Specific subject area	Document Summarization - subfield of natural language processing/computational linguistics/human language technology
Type of data	Raw and processed aligned and unaligned text (UTF8). The dataset contains the following types of data: – **Processed**: – tab-separated value (.tsv) files split into – With summaries: **train, test**, and **dev** [column names: id, type, year, input (judgment), output (summary)] – Without summaries: **all_data** [column names: id, type, year, input (judgment)] – JavaScript Object Notation (.json) files: – **Raw**: zipped portable document format (.pdf) files arranged into folders: with and without summaries → type of judgment (electoral and non-electoral) → each judgment (with or without summary file) in a folder labeled with the judgment name. – **Python files**: .py files containing codes that we used to (1) extract the raw files, (2) transform the pdf files into the processed tsv files, and (3) describe the formatted data.
Data collection	The dataset was collected by scrapping the judgments webpage of the Supreme Court of Appeal of South Africa website. The collected judgments and summaries were each in a portable document format, making their automatic extraction more challenging. We extracted each judgment document automatically and reviewed a random sample to ensure accuracy and completeness. We paired the judgments with summaries and saved them with other identifying metadata. For the judgments without summaries, we extracted them to a separate file along with their metadata. We provide both the curated and raw datasets for reproducibility and other analysis.
Data source location	**Institution:** South Africa Supreme Court of Appeal website **Location:**https://www.supremecourtofappeal.org.za/index.php/component/jdownloads
Data accessibility	**Repository:** University of Pretoria Research Data (Figshare), GitHub, and Huggingface **DOI:** **Huggingface**: 10.57967/hf/3565 **UP Data**: 10.25403/UPresearchdata.27371520.v2 **Direct URL to data:** **Huggingface**: https://huggingface.co/datasets/dsfsi/zasca-sum GitHub: https://github.com/dsfsi/zasca-sum **UP Data:**https://researchdata.up.ac.za/articles/journal_contribution/ZASCA-Sum_a_dataset_of_the_South_Africa_Supreme_Court_of_Appeal_judgments_and_media_summaries_for_legal_documents_summarization_research/27371520 **Instructions for accessing these data**: open access
Related research article	**None**

## 1 Value of the Data

The following are the values of the presented dataset:

• The dataset enables localizing general and legal summarization models into the South African legal domain, improving their performance in South African legal cases. This is the first dataset on the continent, to the best of our knowledge.
• The media summaries, crafted by legal experts, provide concise and accurate reporting, allowing models to learn how to condense complex legal language while retaining critical details.
• The dataset's parallel component is ideal for supervised learning. It offers a benchmark for building new models and evaluating the effectiveness of and adapting current summarization models in the legal domain.
• The unaligned component of the dataset can be used for unsupervised learning, enabling researchers to explore novel approaches to legal summarization without relying on labeled data.
• The dataset, written in South African English, is also a valuable resource when training or localizing other Natural Language Processing (NLP) models to generate content in this dialect.

## 2 Background

Legal text summarization is a challenging task that involves compressing long legal documents into concise summaries while preserving key facts and legal reasoning [1]. These judgments are often detailed, sometimes spanning thousands of words, making them difficult for non-experts to understand. Without layman summaries, it becomes harder for the public to grasp court decisions. The media also faces challenges in accurately reporting these judgments without such summaries. A good legal summary must strike a balance between simplicity and accuracy, ensuring that key aspects of the judgment are conveyed clearly without oversimplifying the legal reasoning behind the decision. Several datasets and models have been developed to support automatic summarization. Examples of such datasets include the US litigation releases^1^, and datasets from the Indian and UK Supreme Courts [1]. Additionally, summarization models like the Longformer Encoder-Decoder (LED) [2] and Pegasus [3] have been adapted^2^^,^^3^ to aid legal document summarization. However, none of these resources cover any country in Africa.

**Fig. 1:**
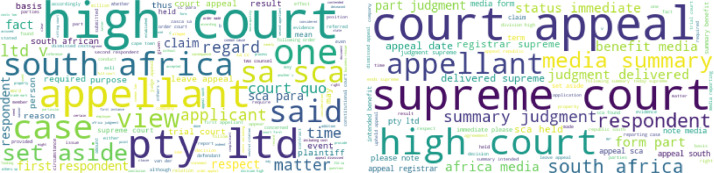
Word clouds generated from the judgments and summaries respectively.

In this work, we present a new dataset of judgment-summary pairs of the South African Supreme Court of Appeal (SCA), called ZaSCA-Sum. The SCA plays a crucial role in interpreting and applying the law in South Africa. Its judgments are often accompanied by layman summaries designed for media reporting. The dataset was collected from the Supreme Court’s website^4^, extracted into readable and structured format for downstream applications such as natural language processing (NLP) or text analysis. The dataset is made of electoral and non-electoral decisions and is suitable for text summarization, and other applications such as model adaptation and other text-intensive tasks. The word clouds in Fig. 1 illustrate the domain of the ZaSCA-Sum dataset. The most relevant words in the dataset are high court, South Africa, court, appeal, supreme court, appellant, claim, respondent, summary judgment, media summary, case, applicant, set aside, pty (proprietary), and ltd. To the best of our knowledge, this dataset is the first of its kind on the African continent.

## 3 Data Description

**Fig. A.1:**
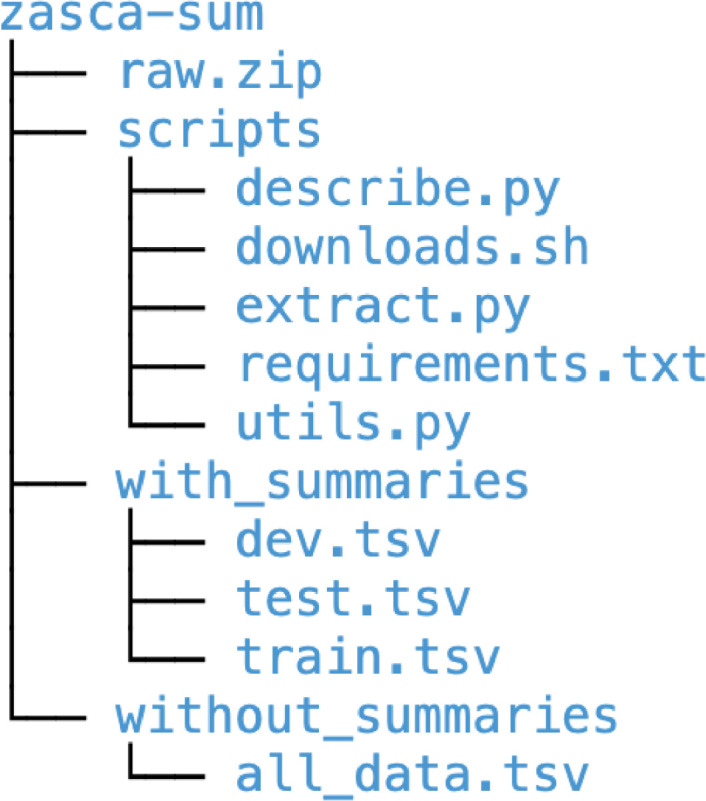
The directory structure of the ZaSCA-Sum dataset.

**Fig. A.2:**
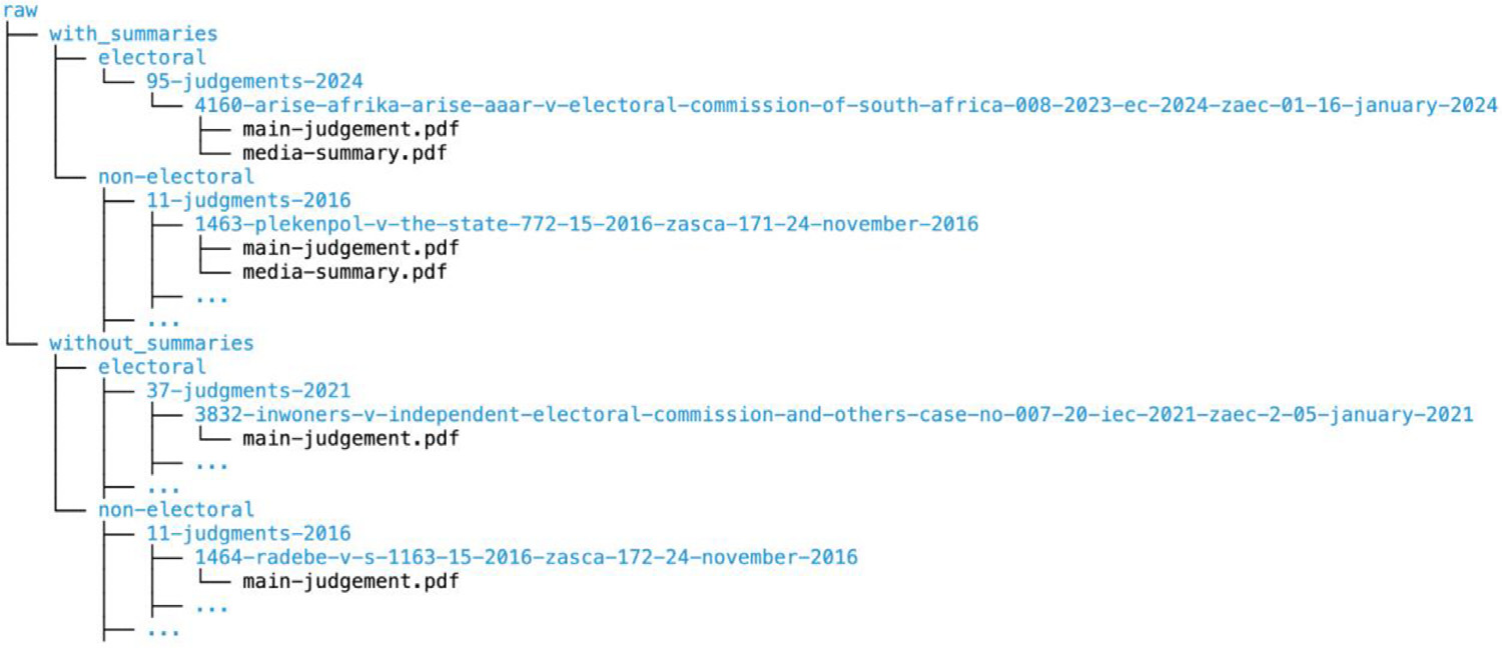
The directory structure of the unzipped raw data folder in the ZaSCA-Sum dataset.

The dataset and accompanying scripts are arranged as subfolders in a folder labeled: “zasca.” Snapshots of the file and folder structure are presented in Fig. A.1, Fig. A.2. The structure is explained as follows: The processed data is in the first two folders as follows, then followed by the zipped raw files and the scripts folders.

• **With_summaries**: This directory contains all the judgments that have been processed and the accompanying summaries. This data component is split into 3 tab-separated value (.tsv) files named – **train.tsv, dev.tsv** and **test.tsv** representing train, validation, and test files, respectively. The tsv files have the id, judgment type, judgment year, judgment text (input), and summary text (output) as column names.
• **Without_summaries**: This directory contains all the processed standalone judgments without accompanying summaries. This data component is contained in one file named **all_data.tsv** and has the id, judgment type, judgment year, and judgment text (input) as column names.
• **Raw.zip**: this is a zip folder containing all the raw files in portable document format (pdf). The folder has subdirectories as follows:
  ○ **With_summaries**: This directory contains all judgments that have summaries. This folder contains two subfolders named electoral and non-electoral, indicating the type of judgments as such. Each subfolder contains a list of the judgment and summary folders labeled by year_id-judgments-year, e.g., 95-judgments-2024. Contained within each judgment year subfolders are folders labeled by the judgment names. A 150-character length restriction was used, and the words in the judgment name were concatenated by ‘-’ to form the name string. Each of these folders contains two pdf files labeled as main-judgement.pdf and media-summary.pdf.
  ○ **Without_summaries**: similar directory structure as with_summaries, except that each of the last folders contains only one pdf file labeled as main-judgement.pdf.
• **Scripts**: these code files are used in processing the raw data, and they are explained as follows:
  ○ **describe.py**: python script that computes the statistical analysis of the processed dataset.
  ○ **downloads.sh**: download the GloVe embeddings for data analysis.
  ○ **extract.py**: python script that extracts the zipped files and folders and extracts and processes the text from the judgment and summary pdf files into the final tsv files contained within the processed folder above.
  ○ **requirements.txt**: a text file containing all the required library dependencies to run the Python scripts.
  ○ **utils.py**: a Python utility script containing all the classes and functions needed for data extraction and loading, file management, and text processing and analysis.

**Fig. B.1:**
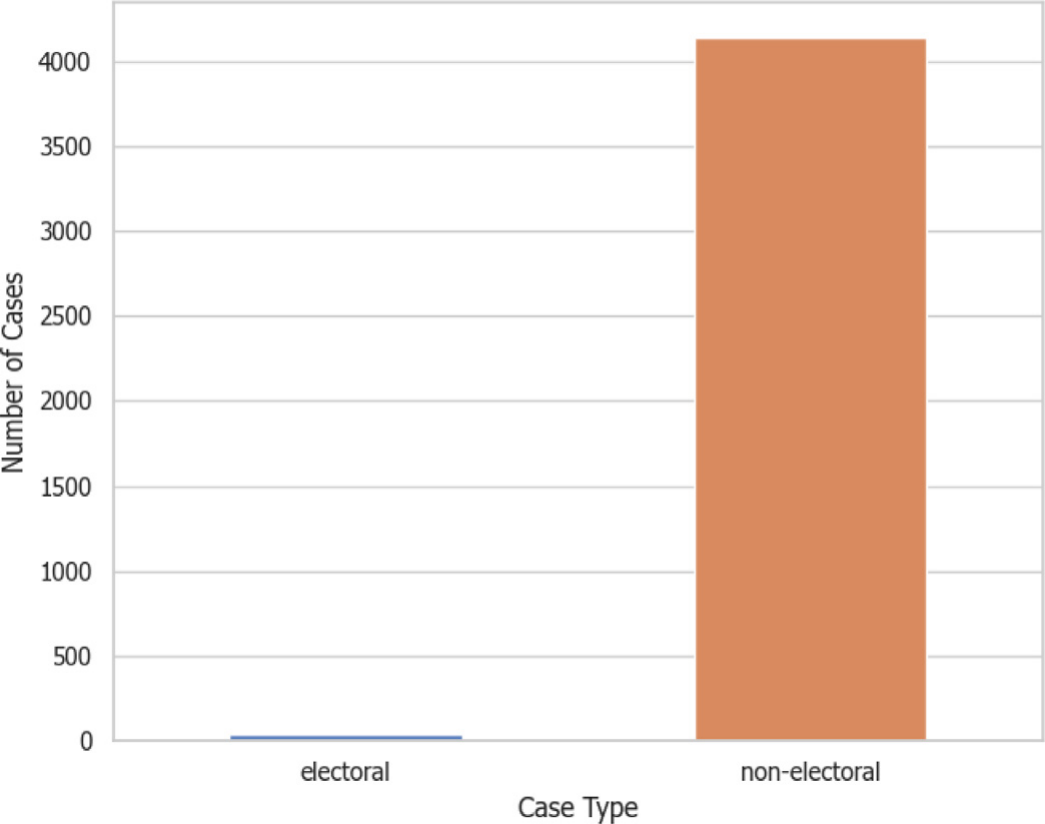
Judgment-type distribution.

The ZaSCA-Sum dataset consists of 4171 judgments, with about 51 % (or 2118) containing a corresponding media summary. Of these, 99% of the data are generic (non-electoral) with the other 1% making up the electoral cases. Fig. B.1 shows the judgment-type distribution.

Table 1 presents a detailed statistical description of the dataset, showing the sentence-, token-, and character-level statistics, detailing the total counts across all documents, the average, standard deviation, maximum and minimum words and sentences in the documents.

**Table 1:** A detailed description of the statistical information of the collected and processed dataset.

stats	Sentences		Words (tokens)		Characters	
	Judgments	Summaries	Judgments	Summaries	Judgments	Summaries
count	418,327	40,562	13,939,214	1,372,001	60,938,673	6,193,849
mean	197.79	19.18	6590.64	648.70	28,812.61	2928.53
std	159.05	13.61	4,988.11	439.64	21,896.36	1,941.70
min	10	1	420	83	1,942	379
90 %	350.00	35.00	11,633.80	1156.40	51,175.40	5156.40
max	2573	238	73,612	7014	323,723	238

The table shows that the judgments can be as long as 73,612 tokens and the summaries as long as 7014. The major challenge of most deep learning models is their ability to accommodate long sequences [4]. Currently, the state-of-the-art sequence-to-sequence (seq-2-seq) models for automatic summarization can accommodate as long as 16,384 tokens and generate up to 1024 tokens [2,3]. Off-the-shelf, therefore, these models can fully utilize more than 90% of the judgments and just below 90% of the available summaries. With the increasing capacity of large language models (LLMs), the ZaSCA-Sum dataset can be fully utilized for fine-tuning and, in some models, few-shot prompting to generate the target summaries.

**Fig. 2:**
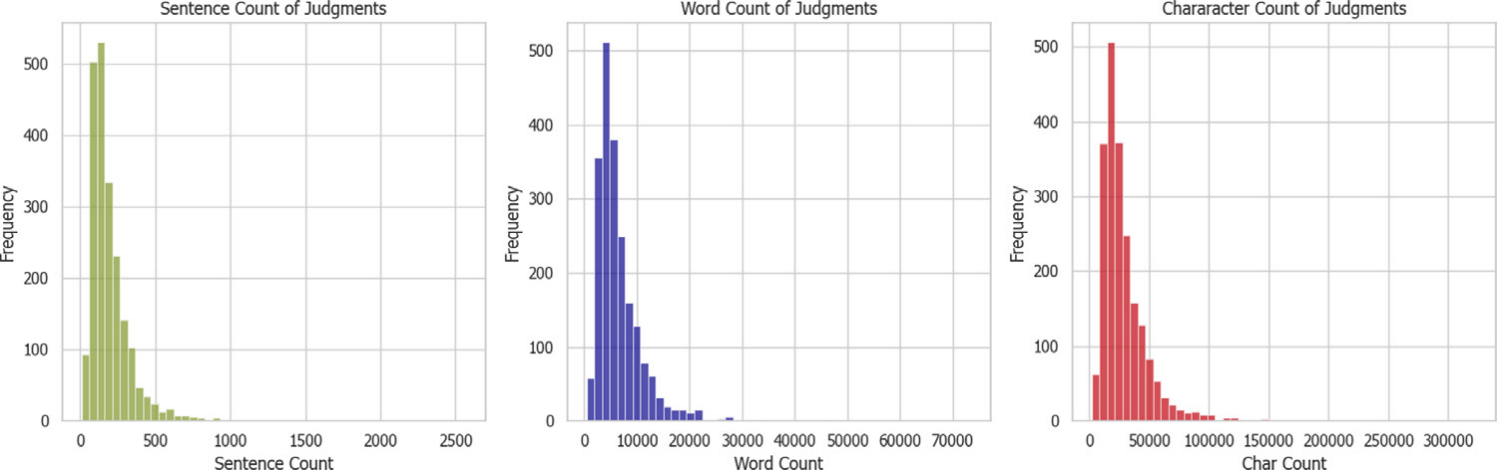
Sentence, word, and character distributions of the judgments.

**Fig. 3:**
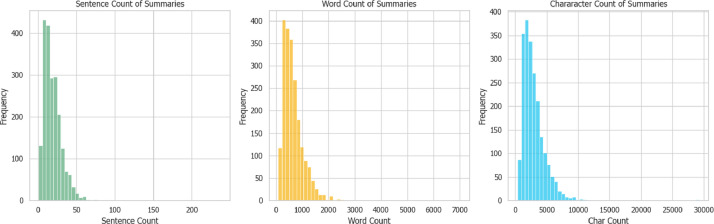
Sentence, word, and character distributions of the summaries.

**Fig. C.1:**
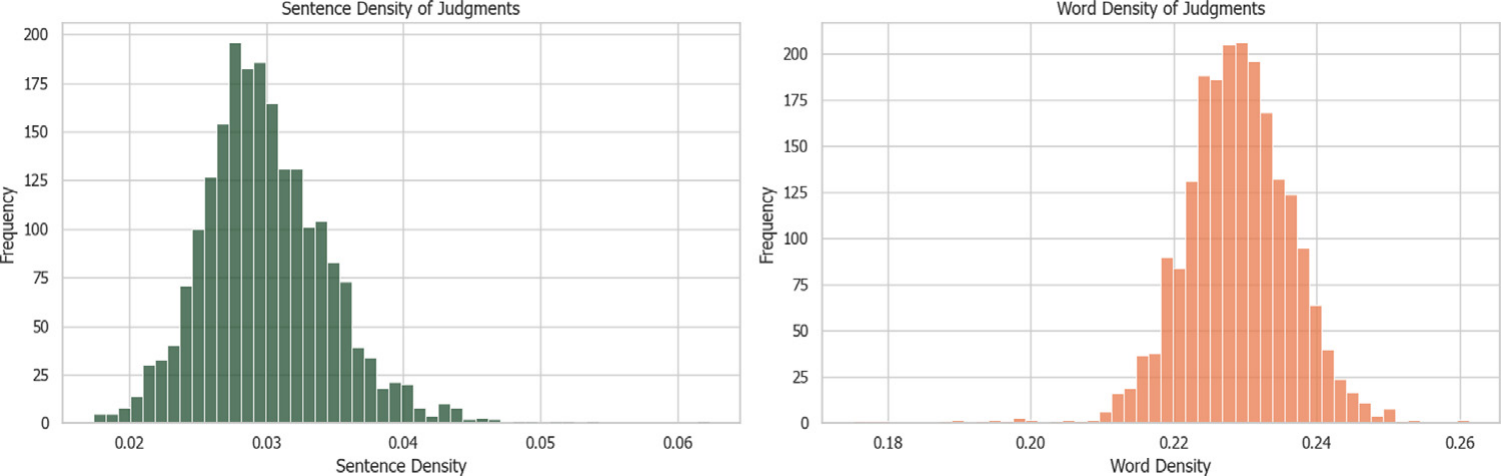
Sentence and word density distributions.

Fig. 2, Fig. 3 show the sentence, word, and character distributions of the judgment and summary texts. It can be observed that the judgments are about 10 times more than the summaries. Fig. C.1 shows the sentence and word density distribution, calculated by dividing each sentence and words by the number of words and characters respectively and averaging. The densities are similar in both the judgment and summary texts.

**Fig. 4:**
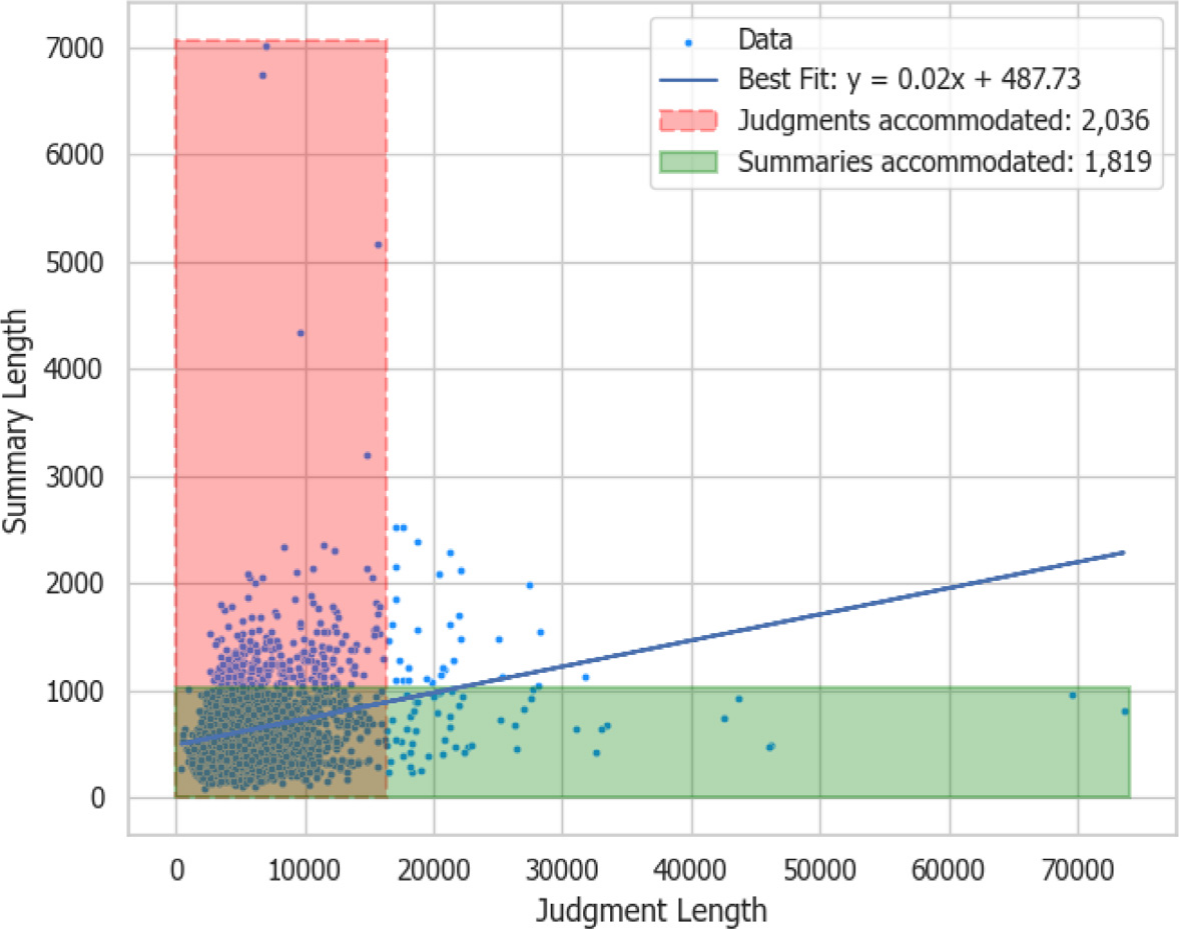
Sentence, word, and character distributions of the summaries.

Fig. 4 shows the difference between the lengths of the judgments and summaries, with rectangular markings showing the judgments and summaries that can be accommodated off-the-shelf by the largest available seq-2-seq models. It also shows the line of best fit (trendline) between the lengths of the judgments and summaries.

**Fig. D.1:**
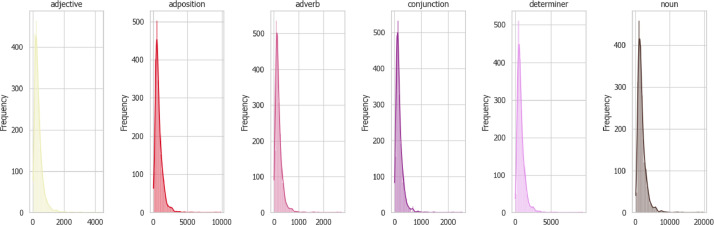
Part of Speech Tags in Judgment Texts.

Finally, Figs. D.1, D.2, D.3, and D.4 show the part of speech tag words, stopwords, and unknown words in the ZaSCA-Sum dataset. Identifying the prevalent stopwords and unknown words gives an understanding of the preprocessing needed in the dataset especially when training machine learning models. We used the NLTK [5] toolkit’s pos_tag function to extract the POS words with the universal tagset [6]. Adjectives such as few, long-standing, and best, adpositions such as in, under, and by, adverbs such as extremely, adequately, and purely, conjunctions such as and, neither, and or, determinants such as a, an, and the, and nouns such as The Supreme Court of South Africa, Roodepoort, November, and Child Care Act are some of the tagged words in the dataset. We used the list of punctuation provided in the Python string library to analyze the punctuation in the dataset, and for the stopwords, we used the English stopwords in NLTK. Stopwords such as the, because, and yourself were found in the dataset. Finally, we used the vocabulary of the GloVe [7] embeddings, containing 400,000 word vectors, to identify unknown words. Most of the identified unknown words are local names and entities such as firstrand, mhlantla, mathopo, and saripa.

**Fig. 5:**
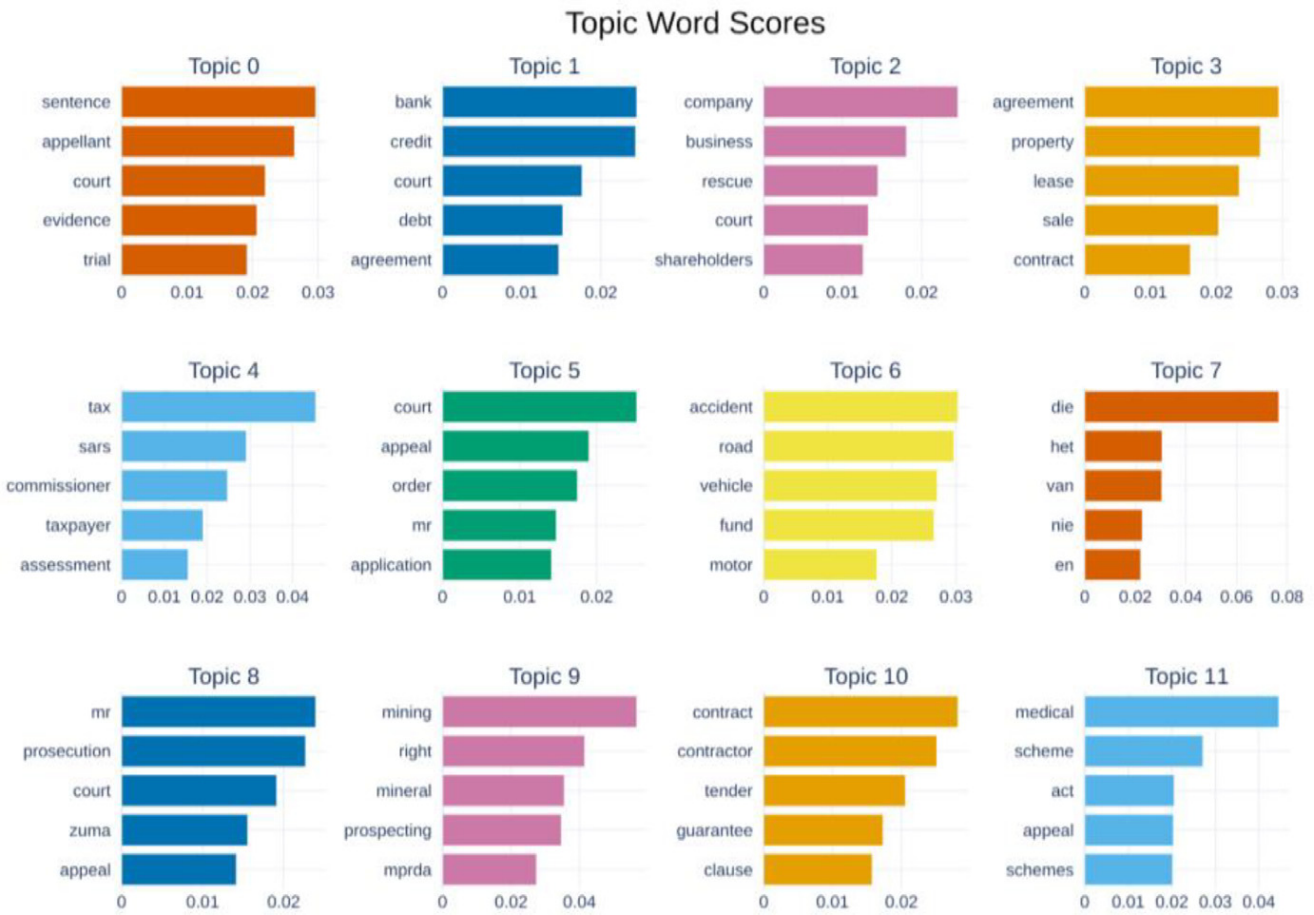
Top 12 topics extracted from combined judgment and summary texts.

**Fig. 6:**
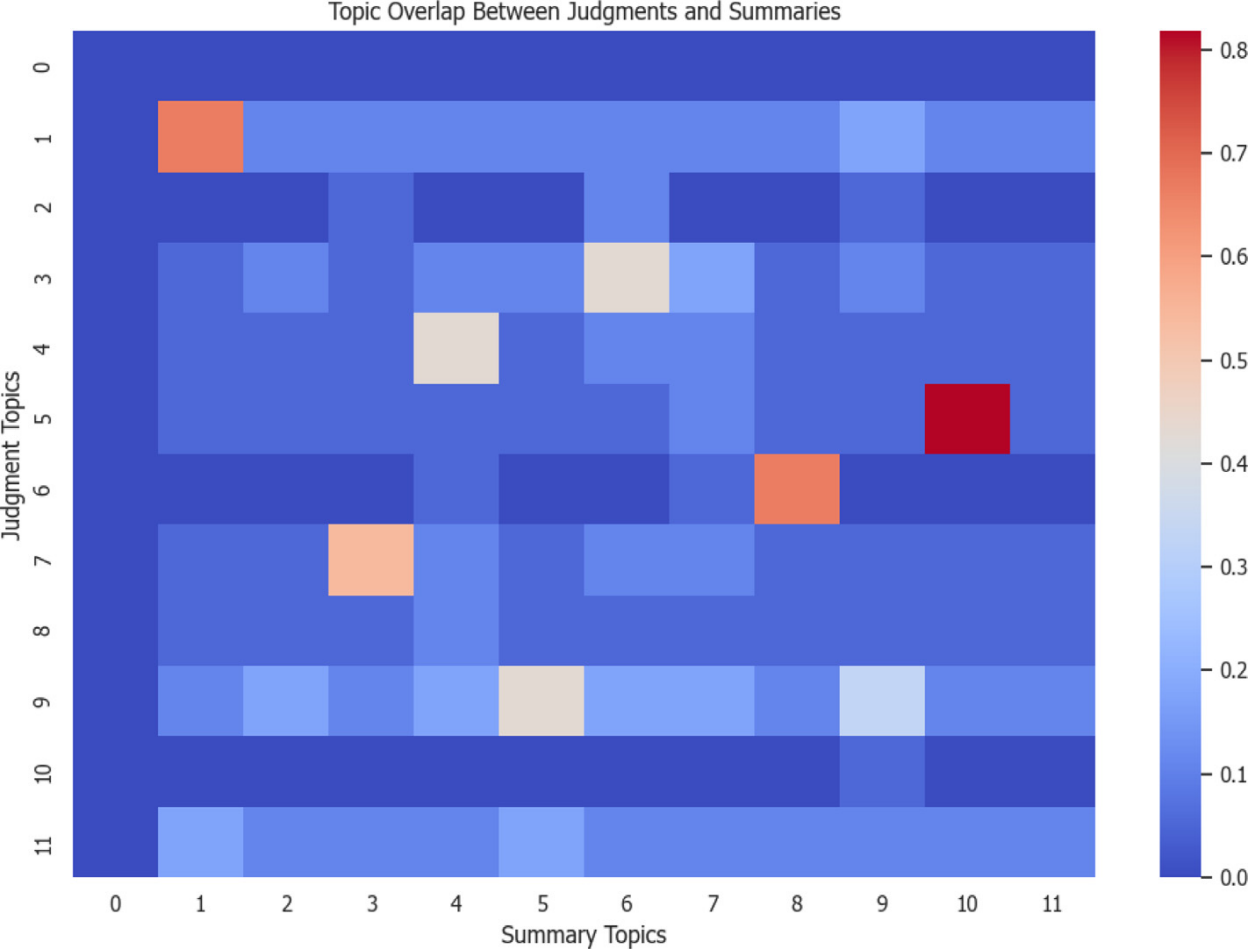
Heatmap showing overlap between the top 12 topics in judgment and summary texts.

**Fig. E.1:**
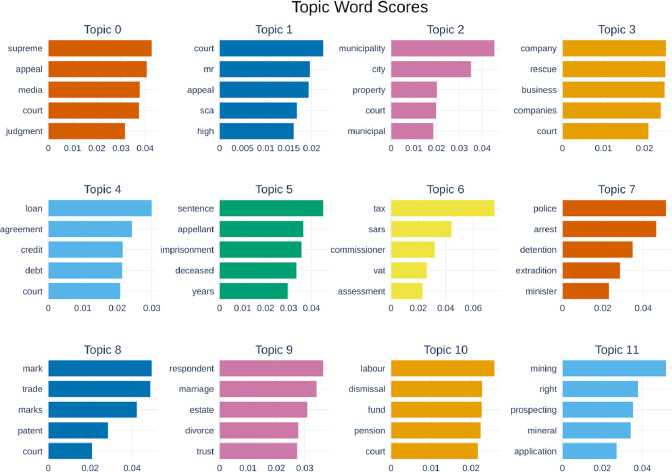
Top 12 topics in judgment texts.

**Fig. E.2:**
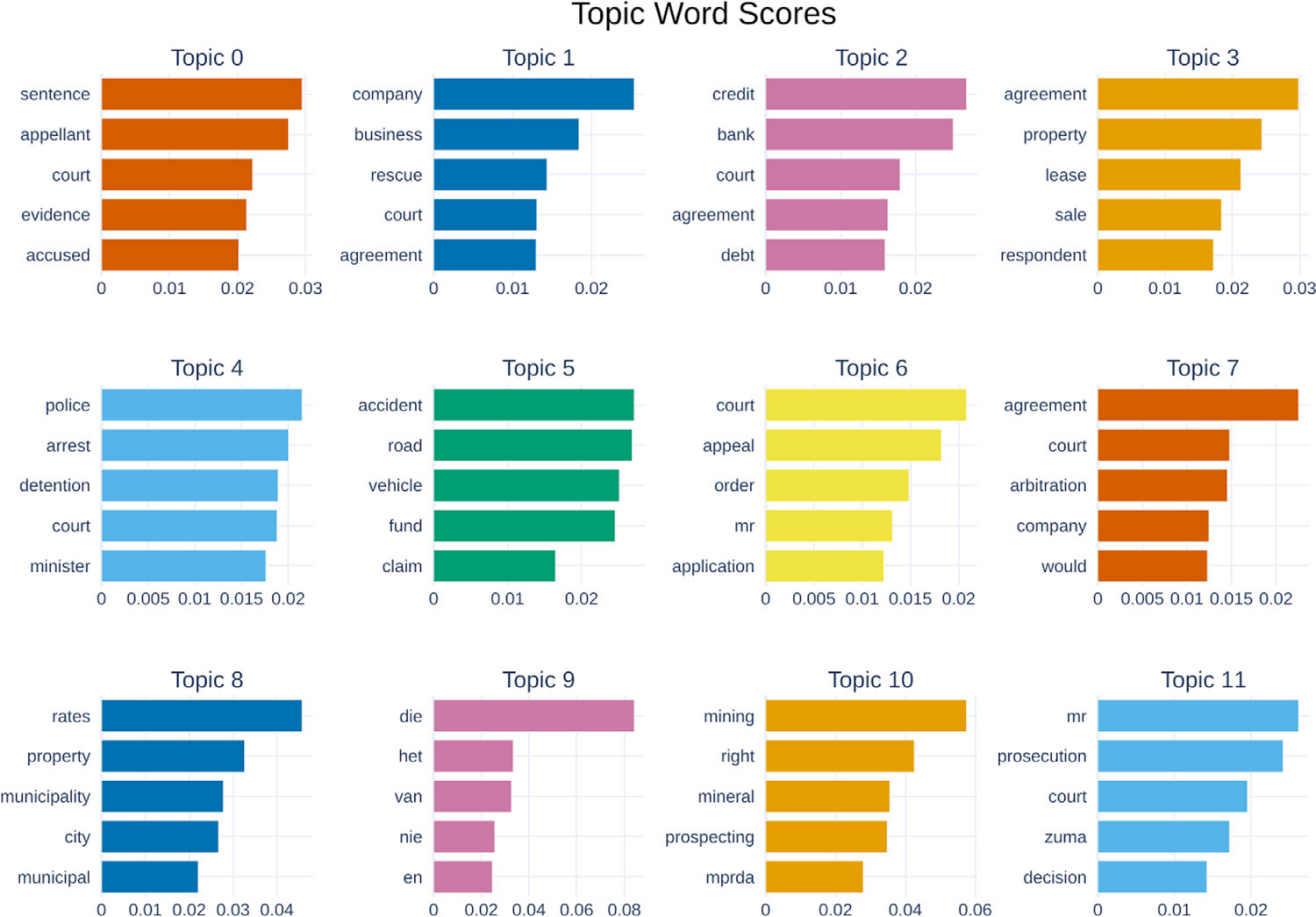
Top 12 topics in summary texts.

Finally, we employed BERTopic, a state-of-the-art topic modeling approach, to uncover meaningful insights within the dataset. This analysis revealed the most relevant topics in both the judgment and summary texts, see Fig. 5, providing a clear understanding of the key themes present in the data. Common topics include judgment appeal, banks credit and debit agreements, company and business recuse agreements, insurance disputes, Zuma’s prosecution and appeal, etc. Additionally, we generated a heatmap in Fig. 6 comparing the top 12 topics derived from the judgment texts and their corresponding summaries, offering a visual representation of the alignment and differences between these two elements. See Fig. E.1, Fig. E.2 for the actual top 12 topics for comparison.

## 4 Experimental Design, Materials and Methods

**Fig. 7:**
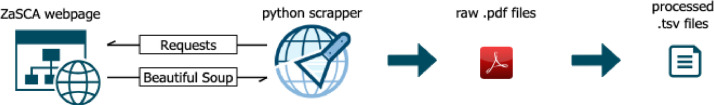
Data collection, extraction, and processing methodologies.

Fig. 7 illustrates the data collection, extraction, and processing processes. The data collection, extraction, and processing scripts are provided in the data repository. The dataset was collected between February 19, 2024, and November 5, 2024.

### 4.1 Data collection

The dataset was collected from the website^5^ of the South African Supreme Court of Appeal (ZaSCA) using a Python script written specifically for this task. The script used the “requests” and “BeautifulSoup” libraries to extract the available judgments and summaries (if present) from each judgment page.

The process began by sending an HTTP GET request to each of the electoral^6^ and non-electoral^7^ base URLs to fetch the pages’ HTML contents. The text response was then parsed using BeautifulSoup with the LXML parser to enable structured navigation of the HTML document. The find_all method was then used to locate all <div> elements with the class ``jd_categories_title_v46'', returning a list of these elements. For each div in the extracted divs, the href attribute of the anchor tag (<a>) within the div was collected and the list of the URLs was sorted by year, that was extracted within the links. These are the links of all the available judgment years.

For each extracted link, a folder was created and labeled using the extracted year and year_id. The script then fetches the main page of each year using the requests.get method and parses it with BeautifulSoup. If the page includes pagination, identified by the presence of a <li> element with the class ``pagination-end'', the largest index—indicating the final page of judgments for that year—was extracted. The pages were iteratively accessed using a calculated range of pages (< largest page index), incremented by n = 10 (the highest number of judgments on one page). For each judgment page, the scrapper fetches the page content, and extracts <div> elements with the class ``jd_download_wrapper'', which contain judgment links. From each <div>, the scrapper attempts to extract two links: the main judgment and an optional media summary. Finally, all valid links were saved for files download.

For each valid judgment (and summary) link(s), the script created a folder, named after the judgment. The judgment name was truncated to 150 characters, if longer. It then checked if the main judgment file (main-judgement.pdf) already exists, and if so, it skipped downloading. If unavailable, it downloads the main judgment and saved it as main-judgement.pdf. It also checks if a media summary file exists, and if available, it was downloaded and saved as media-summary.pdf.

The script includes error handling to skip missing URLs, handle unavailable pages, and manage cases where media summaries are not present.

### 4.2 Data extraction and processing

A utility class was designed to extract and clean text from the collected PDF documents. The class uses the PyMuPDF library (fitz) to extract raw text from each page of a PDF and then processes the text to remove irrelevant elements, such as numeric strings or uninformative lines, while reconstructing coherent paragraphs. First, a method in this class is called “extract_text” opens the PDF file and extracts text from all pages. Each page's text is passed through a cleaning pipeline (“_clean_line”), which splits the text into sentences, removes excess whitespace, and filters out unwanted numeric strings (e.g., page numbers or indices) based on a threshold. Once cleaned, the sentences are passed to the “_combine_paragraphs” method, which merges lines that are part of the same paragraph. This is determined by markers such as square brackets containing digits (e.g., [1]), which indicate continuity. The cleaned and reconstructed text is finally joined into a single string, ready for use. To ensure accurate filtering, two helper methods were used: “_is_numeric_string” and “_is_paragraph_marker”. The function “_is_numeric_string” identifies lines containing only numeric values, commonly used for page numbers, and discards them. Note that a line containing any other character other than a numeric value is not discarded. The function “_is_paragraph_marker” checks for specific patterns, such as bracketed numbers, that signal paragraph continuation. By integrating these methods, the utility class was designed to streamline the process of converting the PDF text of the judgments and summaries into a clean, human-readable format. The extracted texts along with their associated metadata are saved to a .tsv file.

## Limitations

The dataset is limited in size. South Africa is a low-resource country in terms of NLP resources. All indigenous languages and South African dialects of foreign languages are not well represented in available NLP datasets and models. Therefore, even though the dataset is much smaller than available document summarization datasets, it is still a significant amount of data. Also, other English models can easily be adapted to cater for the South African dialect and legal domains. Furthermore, the dataset was collected from the Supreme Court of Appeal website, and more content is still being uploaded, making the data potentially high resource in the future.

## Ethics Statement

The authors have read and followed the ethical requirements for publication in Data in Brief and confirmed that the current work does not involve human subjects, animal experiments, or data collected from social media platforms. The source data is a public document and is exempt from copyright protection: in accordance with Section 12(8)(a) of the South African Copyright Act (Act No. 98 of 1978), no copyright shall subsist in official texts of a legislative, administrative, or legal nature, official translations of such texts, speeches of a political nature, speeches delivered in the course of legal proceedings, or in news of the day that are mere items of press information. This provision ensures that court judgments and similar legal documents are part of the public domain and may be freely reused for academic and research purposes^8^

## Credit Author Statement

**Idris Abdulmumin:** Conceptualization, Methodology, Software, Writing - Original Draft. **Vukosi Marivate:** Supervision, Writing - Original Draft.

## Figures and Tables

**Fig. D.2:**
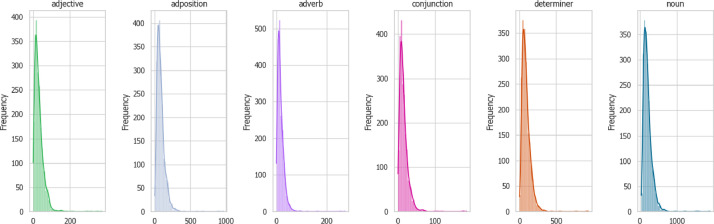
Part of Speech Tags in Summary Texts.

**Fig. D.3:**
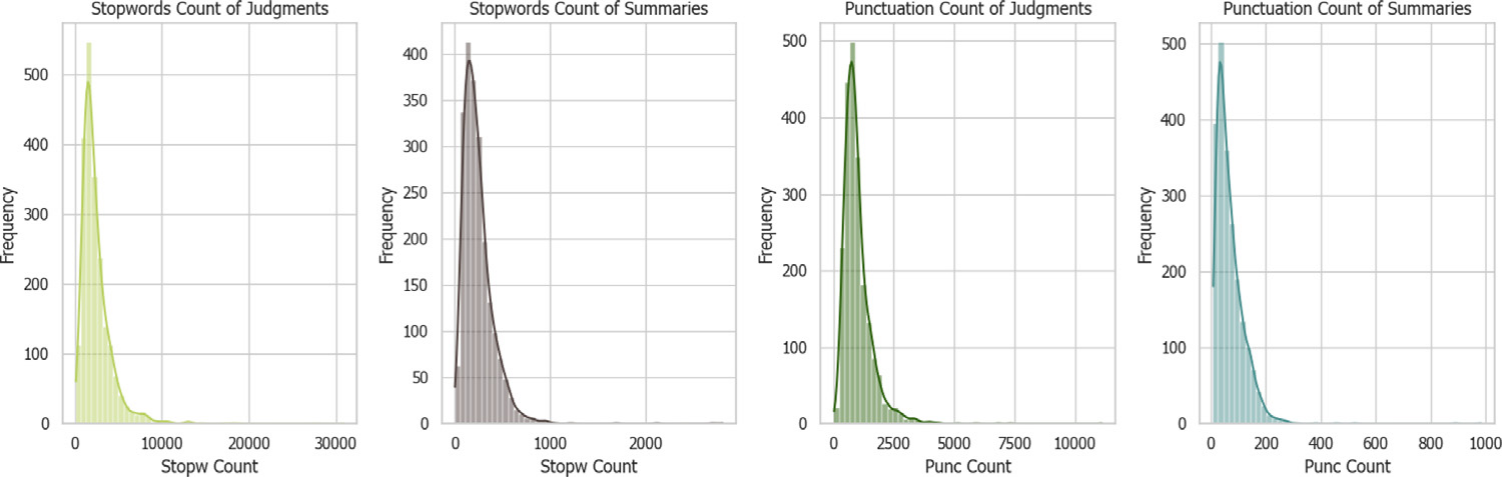
Stopwords in judgment and summary texts.

**Fig. D.4:**
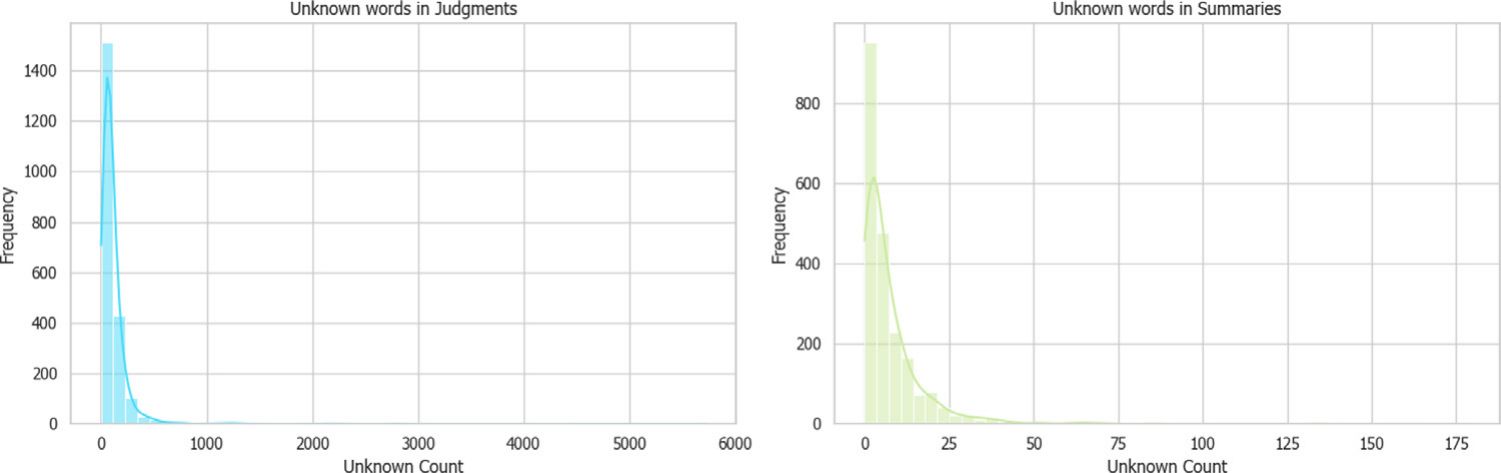
Unknown words in judgment and summary texts.

## Data Availability

HuggingfaceZASCA-Sum: A Dataset of the South Africa Supreme Courts of Appeal Judgments and Media Summaries for Legal Documents Summarization Research (Original data).

## Appendix

**A – Dataset Directory Structure**

**B – Distribution of Judgment Types**

**C – Judgments and Summaries Density Distribution**

**D – Unknown words, Stop words, Punctuation and Part-of-Speech Tags**

**E – Topics in Judgments and Summaries extracted by BERTopic**

## Footnotes

1 https://www.sec.gov/enforcement-litigation/litigation-releases.

2 https://huggingface.co/nsi319/legal-pegasus.

3 https://huggingface.co/nsi319/legal-led-base-16384.

4 https://www.supremecourtofappeal.org.za/index.php/component/jdownloads. Accessed between February 19, 2024, and November 5, 2024.

5 https://www.supremecourtofappeal.org.za/index.php/component/jdownloads.

6 https://www.supremecourtofappeal.org.za/index.php/judgements/electoral-court-judgments/category/36-electoral-court-judgments.

7 https://www.supremecourtofappeal.org.za/index.php/judgements/category/4-judgments.

8 https://www.gov.za/sites/default/files/gcis_document/201504/act-98-1978.pdf.
